# From Triboelectric Nanogenerator to Hybrid Energy Harvesters: A Review on the Integration Strategy toward High Efficiency and Multifunctionality

**DOI:** 10.3390/ma16196405

**Published:** 2023-09-26

**Authors:** Yifei Wang, Ning Wang, Xia Cao

**Affiliations:** 1 Center for Green Innovation, School of Mathematics and Physics, University of Science and Technology Beijing, Beijing 100083, China; d202210430@xs.ustb.edu.cn; 2 Beijing Institute of Nanoenergy and Nanosystems, Chinese Academy of Sciences, Beijing 100083, China

**Keywords:** hybrid energy harvesters, performance improvement, triboelectric nanogenerators, multifunction

## Abstract

The rapid development of smart devices and electronic products puts forward higher requirements for power supply components. As a promising solution, hybrid energy harvesters that are based on a triboelectric nanogenerator (HEHTNG) show advantages of both high energy harvesting efficiency and multifunctionality. Aiming to systematically elaborate the latest research progress of a HEHTNG, this review starts by introducing its working principle with a focus on the combination of triboelectric nanogenerators with various other energy harvesters, such as piezoelectric nanogenerators, thermoelectric/pyroelectric nanogenerators, solar cells, and electromagnetic nanogenerators. While the performance improvement and integration strategies of HEHTNG toward environmental energy harvesting are emphasized, the latest applications of HEHTNGs as multifunctional sensors in human health detection are also illustrated. Finally, we discuss the main challenges and prospects of HEHTNGs, hoping that this work can provide a clear direction for the future development of intelligent energy harvesting systems for the Internet of Things.

## 1. Introduction

With the rapid development of the Internet of Things and artificial intelligence, various portable and wireless electronic products and their derived intelligent sensor systems are widely present in our life [1,2,3,4,5]. Due to the complex and variable nature of the application scenarios [6,7], intelligent systems may require a large number of sensors to provide support, where the issue of energy supply becomes a challenge because the long-term operation of the system are the basis to fully realize their power [8]. In such systems, electronic products and smart sensors are small and portable [9,10,11]. Traditional power sources such as electrochemical batteries are no longer able to meet the requirement of such electronic products and systems because of the limited volume capacity and safety risks [12,13].

Nowadays, various energy harvesting technologies have been developed, such as triboelectric nanogenerators (TENGs) [14,15,16], piezoelectric nanogenerators (PENGs) [17], electromagnetic generators (EMGs) [18], thermoelectric/pyroelectric generators (PyENGs/TEGs), and solar cells (SCs) [19,20]. Among these energy harvesters, TENGs are invented on the basis of the triboelectric effect and electrostatic induction coupling, and are considered as promising for powering potable electronics and sensor nodes, as well as for having a wide material selection, being simple to manufacture, and being lightweight [21,22,23].

However, to meet the increasing power consumption requirement of more powerful electronics, it is anticipated that TENGs will be integrated with other energy harvesting devices to build hybrid energy harvesters so that multiple energy sources can be simultaneously collected efficiently from the surrounding environment [24,25,26,27]. Since Wang et al. proposed hybrid nano-energy harvesting systems for mechanical and solar energy harvesting in 2009 [28,29,30], this multi-effect coupling strategy has been extended to different types of energy harvesting systems, and their integration also presents multifunctional characteristics [27,28,31,32,33,34].

**Figure 1:**
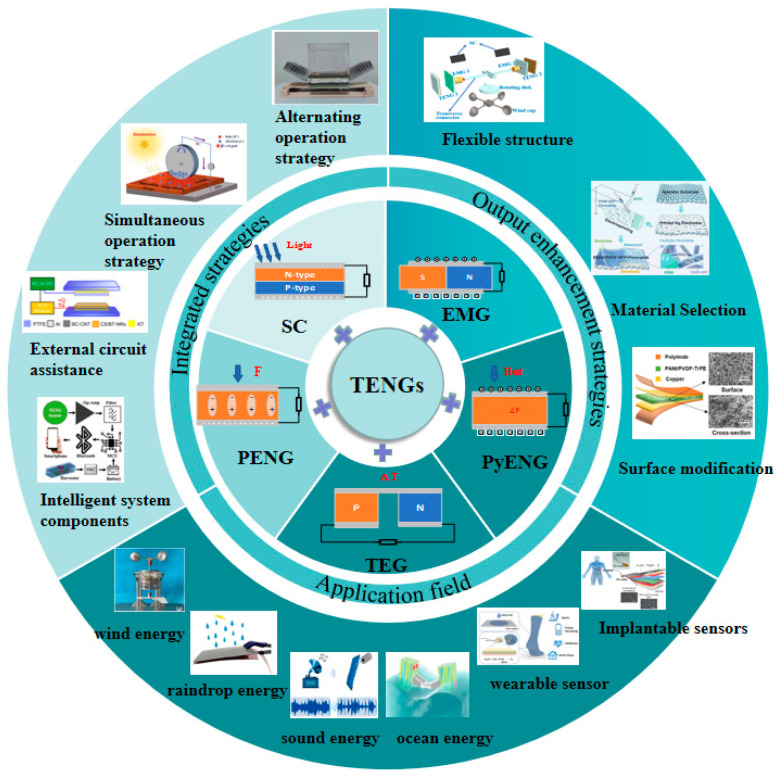
Recent progress of HEHTNGs.

In this article, the latest research progress of HEHTNGs and the working principles of different devices are systematically reviewed (Figure 1). Key strategies that are developed to improve the performance and expand integration concepts of HEHTNGs are emphasized. To provide readers with more information, a clearer view, and analysis, the latest applications of HEHTNGs as multifunctional sensors in human health detection are also introduced. Finally, the main challenges and prospects for the future development of HEHTNGs are pointed out in terms of interface modification, structure optimization, material selection, and intelligent system integration.

## 2. Working Principle of HEHTNGs

### 2.1. Mechanical Energy Harvesting

#### 2.1.1. TENG

**Figure 2:**
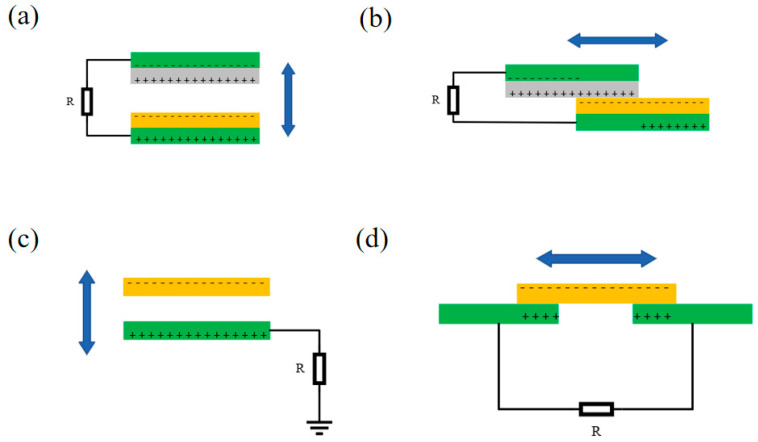
Four fundamental modes of TENGs: (**a**) vertical contact-separation mode, (**b**) lateral sliding mode, (**c**) single-electrode mode, (**d**) freestanding triboelectric layer mode.

TENGs can effectively convert mechanical energy into electrical energy based on Maxwell’s displacement current theory [35]. Nowadays, there are four modes of operation of TENGs: contact separation mode, lateral sliding mode, single electrode mode, and friction independent mode (Figure 2) [36]. The triboelectric effect is a phenomenon where materials with different electron affinities generate triboelectric charges during contact detachment, which may flow in an external circuit to form an electric current where the electric induction effect is coupled [37,38]. By periodically making contact and separating, a large amount of charge can then be generated, and the resulting charge can be harvested for energy by a rectifying circuit. Many materials have a triboelectric effect during the contact-separation process, so there is a wide choice of triboelectric layer materials [39], such as PVC, wool, PDMS, etc. The difference in polarity of different materials reflects the different affinities of the materials for electrons [40,41], and materials with a large difference in polarity should be used for the preparation of high-output TENGs so that the triboelectric effect is also stronger [42].

#### 2.1.2. PENG

**Figure 3:**
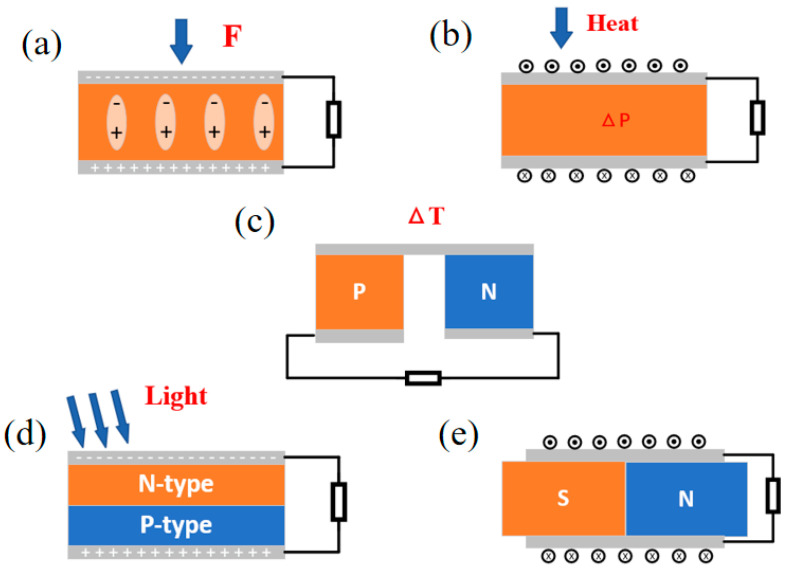
Schematic diagram of device working principle: (**a**) PENG, (**b**) PyENG, (**c**) TEG, (**d**) SC, (**e**) EMG.

The basic principle of PENGs is to use the positive piezoelectric effect of the piezoelectric material (Figure 3a). The positive piezoelectric effect is where when the piezoelectric material is subjected to an external force, the disordered electric dipoles in the material are reversed to form a regular and sequential arrangement, and the centers of the positive and negative charges move toward the opposite surfaces of the material, forming a certain potential difference [43,44,45]. This external force can come from human motion, vibration, noise, etc. In fact, the piezoelectric effect is a coupling phenomenon between mechanical and electrical states. Since PENGs were first proposed by Wang Zhong Lin et al. in 2006 [46], many different structures and materials of PENGs have appeared to prepare an efficient energy harvesting device [47]. Nowadays, inorganic materials such as piezoelectric ceramics and crystals (Quartz, ZnO, AlN, PZT, and BTO) have disadvantages such as hardness, and the focus of research has shifted toward the polymer polyvinylidene fluoride (PVDF) fiber/film and its copolymers, which have extended the application of PENGs to a variety of energy harvesting and wearable electronic devices due to their good flexibility [48,49,50].

### 2.2. Thermal Energy Harvesting

#### 2.2.1. PyENG

In general, the harvesting of thermal energy relies mainly on the Seebeck effect (Figure 3b), but if there is no spatial gradient when the temperature changes with time, the Seebeck effect cannot collect thermal energy. In this case, the pyroelectric effect can be effective in harvesting waste thermal energy. PyENGs work by exploiting the pyroelectric effect of a material, which refers to the release of charge as the polarization strength changes with temperature, where a change in temperature causes a voltage or pyroelectric current to be generated at both ends of the material until a new equilibrium is reached [51,52]. As most piezoelectric materials also have pyroelectric properties, the pyroelectric effect is similar to the piezoelectric effect, which is also a natural physical effect of crystals. Thus, based on the simultaneous existence of piezoelectricity and pyroelectricity, the same material can acquire both mechanical and thermal energy in different application scenarios. Piezoelectric materials with spontaneous polarization such as P(VDF-TrFE), PZT, ZnO, CdS, and KNbO_3_ are often used to make pyroelectric generators. By harvesting waste heat energy, pyroelectric nanogenerators can be used in applications such as temperature monitoring and healthcare [53,54].

#### 2.2.2. TEG

Waste thermal energy is one of the most easily overlooked energy sources. With the deepening of research, thermal energy harvesting can be applied to various aspects, among which not only can PyENGs collect thermal energy but TEGs can also convert thermal energy into electrical energy under very small temperature differences. The working principle of TEGs is the thermoelectric effect (Figure 3c), which refers to the movement of electrons and holes inside certain objects when heated, resulting in the generation of current or charge accumulation [55,56]. TEGs have a simple, environmentally friendly, safe, and reliable structure, as they do not include mobile mechanical components and are mainly composed of N-type and P-type semiconductor material units connected in electrical series and thermal parallel. In the presence of temperature differences, these two types of materials will generate electrons and holes, forming an accumulated potential, resulting in a potential difference that leads to the occurrence of current. Therefore, connecting different thermoelectric materials in series is one of the ways to improve the output performance of TEGs, and the selection of thermoelectric materials is also one of the ways. The performance coefficient ZT is the standard for measuring the thermoelectric performance of materials. Common Bi_2_Te_3_ materials with high conductivity and low thermal conductivity after doping are widely used. In addition, some materials such as PbTe, CoSb_3_, and Mg_2_Si have also been reported for the production of TEGs [57,58,59,60].

### 2.3. Solar Energy Harvesting

#### Solar Cells

Solar energy is the most important renewable energy source, and is now applied to solar heating, photosynthesis, the PV effect, and other aspects. Solar cells are a device that uses chemical and physical phenomena to convert light energy into electricity [61,62]. The working principle of SCs is based on the photovoltaic effect (Figure 3d). The device is composed of N-type and P-type semiconductors internally, and PN nodes are formed at the contact surface of N-type and P-type semiconductors. When the device is illuminated, carriers are generated internally due to the concentration effect, and electrons and holes move to form a current. Then, electricity can be collected through electrode transmission [63,64,65]. Generally speaking, solar cells are divided into silicon-based solar cells, dye-sensitized solar cells (DSSCs), organic solar cells, and perovskite solar cells [66,67,68]. The conversion efficiency and other parameters of solar cells are influenced by material types, unit structures, manufacturing processes, and other technologies. Therefore, in order to improve conversion efficiency, doping technology and coating technology have become the mainstream research on solar cells [69,70].

### 2.4. Electromagnetic Energy Harvesting

#### EMG

The basic working principle of EMGs is based on Faraday’s law of electromagnetic induction (Figure 3e), consisting of a magnet and a moving coil. The electromotive force is generated in the coil by a time-varying magnetic field or by the movement or deformation of the coil relative to the magnetic field line. The electromotive force of EMGs is mainly determined by the characteristics of the material, device size, device structure, and input mechanical energy [71]. In addition, due to their high energy conversion efficiency and good stability in the high-frequency range, EMGs are widely used in fossil fuels, thermal power, and wind power generation. EMGs can be roughly divided into several types: mechanically into the rotation, linear, or multi-temporal kinetic input, and electrically into direct or alternate current [72,73,74,75,76]. EMGs have low impedance, low voltage, and high current output characteristics compared to TENGs, and their triggering method is similar to TENGs. Therefore, many researchers have combined EMGs with TENGs to form a hybrid system, increasing their application range.

## 3. Output Enhancement Strategies for Hybrid Energy Harvester

From TENGs to hybrid energy harvesters, the high efficiency and versatility of devices are closely related to structural design, material selection, and surface modification [77]. A reasonable structural design facilitates device integration and adapts to complex environmental applications [78,79]. Moreover, material selection and surface modification are still the main methods for improving the output in order to achieve multiple-effect coupling.

### 3.1. Flexible Structure

**Figure 4:**
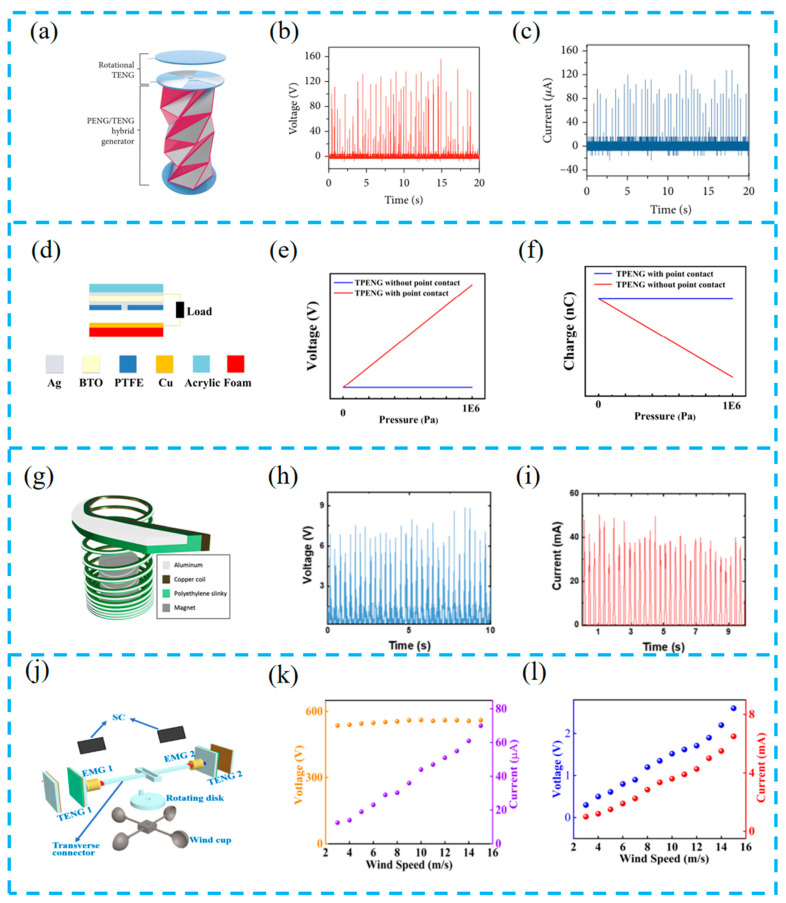
(**a**) TCO-HG composed of rotational triboelectric nanogenerator (TENG) and piezoelectric nanogenerator (PENG)/TENG hybrid generator; (**b**) open-circuit voltage and (**c**) closed-circuit current output of the TCO-HG with the rectifier circuit; reprinted from [80] with permission from research. (**d**) Schematic diagram of the TPENG structure; the voltage (**e**) and transferred charge (**f**) for the TPENG with point contact and without point contact under certain pressure by COMSOL software package (https://cn.comsol.com/); reprinted from [81] with permission from Elsevier. (**g**) The schematic illustration of S-TEHG; (**h**) rectified voltage and (**i**) current output; reprinted from [82] with permission from Elsevier. (**j**) Schematic illustration of the local view of the HEHD, which mainly consists of TENGs and EMGs as well as the commercial SCs; (**k**) the corresponding relationship between the V_OC_, I_SC_, and wind speed of the TENG 1; (**l**) dependence of the V_OC_ and I_SC_ of the EMG 1 on the wind speed; reprinted from [83] with permission from Elsevier.

This section focuses on the flexible structure of the hybrid energy harvesting device, and the reasonable structural design enhances the device’s consistency and greatly improves the operating stability and output performance of the device. A hybrid nanogenerator with triangular cylindrical origami was prepared based on ancient origami (Figure 4a–c). The base is pentagonal and the column is composed of multiple vertical TENGs, rotating TENGs, and PENGs. This structural design allows the PENG and TENG to have high mechanical deformation and a large surface area, respectively. After testing, the contact area of the single layer of the column is enlarged by a factor of 1.38, and each generator can produce power output individually [80].

Increasing the contact area is a commonly used method in structural design, in addition to forming fast charge transfer channels to achieve changes in the internal structure of the thin film. Zhu et al. drilled a hole in the friction layer to serve as a conductive channel (Figure 4d–f), allowing for the formation of a metal-to-metal point contact between the two electrodes, indirectly increasing the rate of charge transfer. This unique structure helps to study the interaction between friction and piezoelectric charges by regulating the direction of the ferroelectric polarization of the BTO [81].

Lee et al. proposed a TENG-EMG hybrid generator (S-TEHG) with elasticity (Figure 4g–i), where a polyethylene (PE) spring substrate, an aluminum electrode layer, a copper coil, and a magnet are composed. The spring structure design has an electric multi-peak waveform mechanism, exhibiting continuous wave propagation under a single input. By generating a stable mixed output of 8.87 V (maximum peak voltage) and 49.6 mA (current) through simple hand movements, it can easily be used as a portable power source for devices in daily life [82].

Changing the operating mode of devices through external components can also improve device performance. Wang et al. used lateral connectors to connect a TENG, EMG, and SC to collect wind and solar energy (Figure 4j–l), converting rotational motion into translational motion, changing the operation of the device, reducing wear between the two frictional layers, and greatly improving the durability of the TENG [83].

Due to the flexible structure design of the device, the output performance and stability are improved, but it is easier to neglect other characteristics such as flexibility and biocompatibility [84].

### 3.2. Material Selection

**Figure 5:**
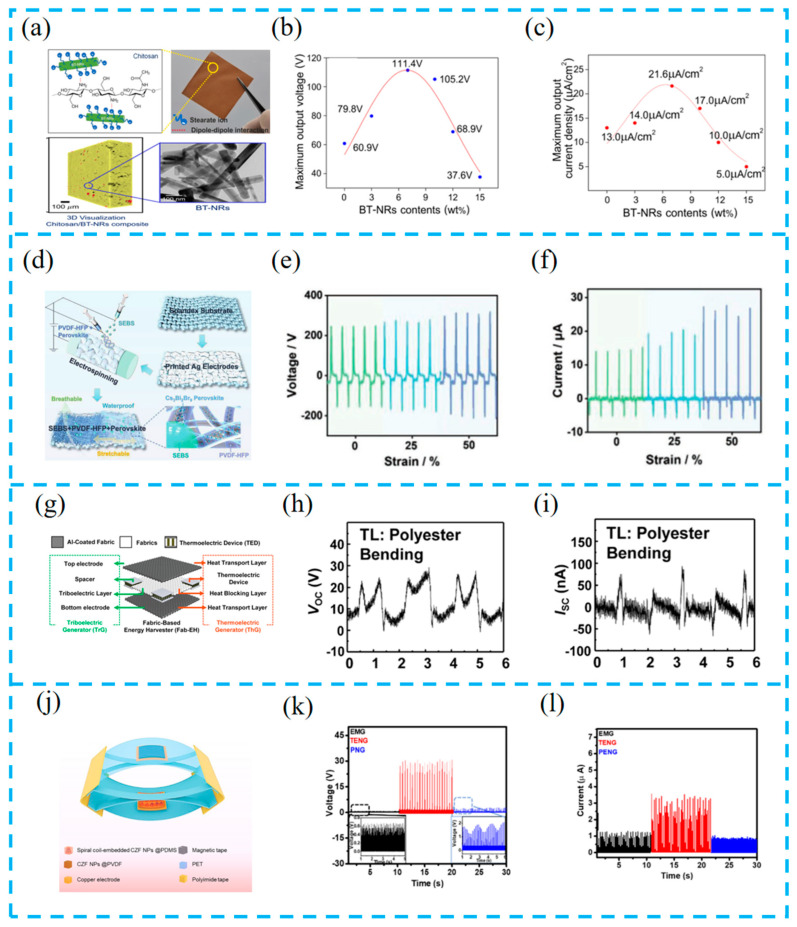
(**a**) Different views of 3D virtualization by SRXTM analysis of CS/BT-NRs 7 wt.% composite film, with an inset of the TEM image for BT-NRs; (**b**) average maximum voltage output and average maximum current output (**c**) with a tendency for varying amounts of BT-NRs fillers (0, 3, 7, 10, 12, and 15 wt.%) and composited films; reprinted from [86] with permission from Elsevier. (**d**) Schematic illustration of the fabrication process of an LPPS-NFC-based TPENG device; (**e**) the output voltage and current (**f**) of LPPS-NFC-based TPENG under 0%, 25%, and 50% strain; reprinted from [87] with permission from Wiley Online Library. (**g**) Schematic illustration of the Fab-EH and a role of each component for TrG (left) or ThG (right) operation; measured (**h**) V_OC_ and I_SC_ (**i**) under bending motion; reprinted from [88] with permission from Elsevier. (**j**) Three-dimensional view of the HNG. Rectified separated (**k**) output voltage and (**l**) output current of each energy harvesting device in the HNG; reprinted from [89] with permission from Elsevier.

It is well known that the selection of a suitable functional layer material is one of the important factors for improving the high-performance output of hybrid energy harvesting [85]. The functional material has different response strengths to different stimulation sources, which leads to its diverse selection and thus makes the hybrid energy harvesting device versatile. For example, Pongampai et al. used chitosan, a natural polymer with naturally degradable, biocompatible, and non-toxic properties, to hybridize with lead-free BaTiO_3_ nanorods to form hybrid films (Figure 5a–c). The large number of hydroxyl (-OH) and amine (-NH_2_) groups in chitosan readily release electrons, thus generating positive zeta potential and enhancing matrix cation capture. The tested power output is more than four times higher than the power enhancement of pristine chitosan TENGs, achieving an ultra-high effective charge density [86].

Meanwhile, perovskite materials have excellent electrical properties, making them a hot research topic nowadays. Jiang et al. prepared a multifunctional nanofiber composite material (LPPS NFC) by electrospinning lead-free perovskite/polyvinylidene fluoride co hexafluoropropylene (PVDF HFP) (Figure 5d–f). As a local nucleating agent, Cs_3_Bi_2_Br_9_ increases the electron capture ability and polar crystalline phase. Forming a good energy level matching with PVDF-HFP improves the electron transfer efficiency and reduces charge loss. The experiment proved that the device can operate for a long time, and it set a record regarding the the output voltage of a halide perovskite-based nanogenerator [87].

In recent years, fabrics have been used as carriers to provide high friction properties through various technologies. Kim et al. reported a Fab-EH-based energy collector (Fab-EH) (Figure 5g–i). The fabric prepared using liquid-phase aluminum (Al) coating technology not only has high friction but also high conductivity and thermal conductivity, effectively collecting mechanical and thermal energy from the human body [88].

The functional layer prepared by Nawaz et al. consists of cubic zinc ferrite nanoparticles (CZF NPs) and a polymer matrix, realizing a multifunctional hybrid multimodal nanogenerator (HNG) (Figure 5j–l). The embedded multi-helix coil structure provides efficient multimodal energy harvesting. At the same time, the device has the characteristics of a high output and low internal resistance and also has three working modes, which provides an idea for achieving a multifunctional hybrid nanogenerator [89].

In the past, it was common to choose environmentally friendly or toxic high-performance materials. With the development of flexible and biocompatible wearables, it is a trend to gradually choose natural cellulose or plant extracts for device preparation [90].

### 3.3. Surface Modification

**Figure 6:**
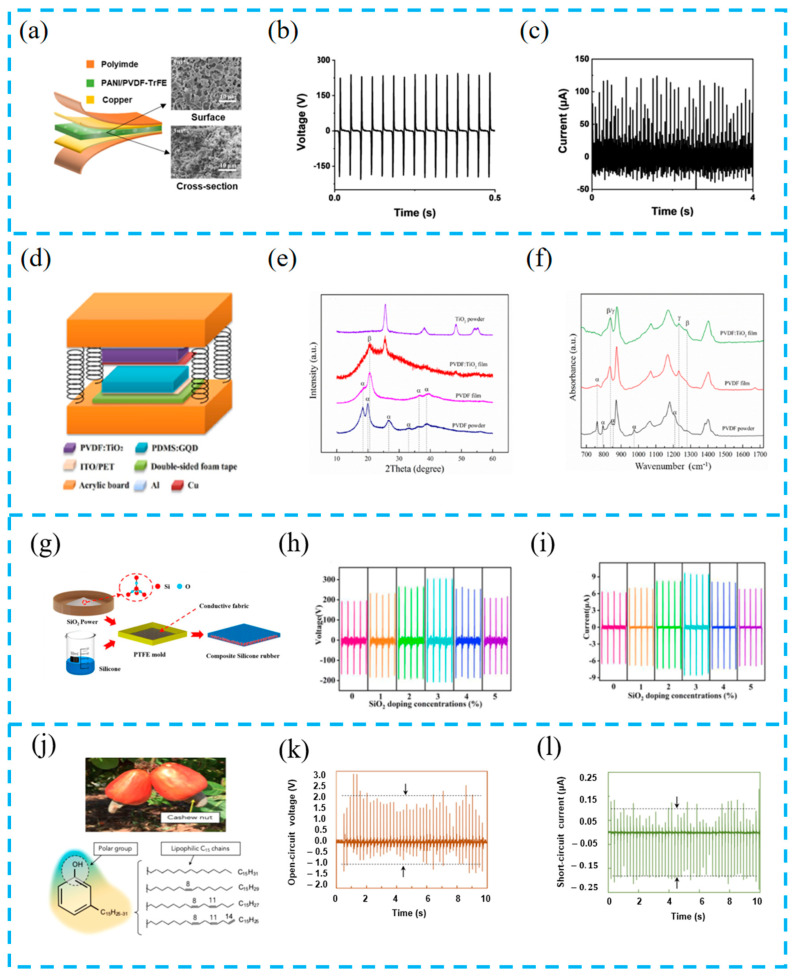
(**a**) Structure diagram of the PTNG and surface and cross-section SEM images of the aerogel bulk; (**b**) the optimal output voltage and (**c**) current of the quenched PANI/PVDF-TrFE PTNG; reprinted from [91] with permission from Elsevier. (**d**) The schematic diagram of PT-NG; (**e**) XRD pattern of PVDF films before and after modification with TiO_2_ nanoparticles; (**f**) FTIR absorption of PVDF films before and after modification with TiO_2_ nanoparticles; reprinted from [92] with permission from Elsevier. (**g**) Schematic of the composite silicone rubber; (**h**,**i**) output performance of composite silicone rubber under different conditions; reprinted from [93] with permission from Elsevier. (**j**) Cashew nut as a source for the production of cardanol oil and chemical structure of cardanol with the indication of the polar group and lipophilic chains; (**k**,**l**) open-circuit voltage and short-circuit current generated by the hybrid PENG under finger tapping (∼2 N, ∼5 Hz) and with a 10 MΩ probe; reprinted from [94] with permission from ACS.

In addition to material selection, the surface modification of hybrid energy harvesting devices is very important. Surface modification technology can change the physical morphology and electrical properties of the functional layer surface, enabling the energy harvesting device to have high-performance output. Yu et al. reported that the use of liquid-nitrogen-induced phase transition and in situ doping methods resulted in a piezoelectric polymer containing 71% β after quenching (Figure 6a–c). Doping sodium carboxymethyl cellulose (SCMC) not only increases the piezoelectric properties of the film but also increases porosity [91].

Using doping technology, the composition of the thin film is changed to affect the electrical properties of the friction layer. Yang et al. modified PDMS and PVDF using graphene quantum dots (GQDs) and titanium dioxide (TiO_2_) nanoparticles (Figure 6d–f). The addition of GQDs improved the electrical properties of the thin film, and TiO_2_ induced PVDF β by increasing the number of phases. This method changes the microstructure of the PDMS surface, increases the effective area contact, and enhances the piezoelectric properties of PVDF during the unpolarization process. Overall, the improved device more effectively converts mechanical energy into electrical energy [92].

Mu et al. proposed a wind energy collector (WEH) composed of an EMG and TENG, (Figure 6g–i). Using doping and surface modification techniques, the addition of SiO_2_ to silicone rubber increased the dielectric constant of the functional layer, and the microstructure prepared increased the contact area between the friction layers. The array contact separation structure was fabricated using modified silicone rubber, a large area curved substrate, and a Lelo triangular cam with local degrees of freedom. After testing, The output of the device doped with modified SiO_2_ is more than double that of the original device [93].

Some inorganic materials are expensive and contain substances that are toxic to the human body. It is a trend to look for low-cost natural modifiers. Mariello et al. prepared a metal-free hybrid piezoelectric nanogenerator (HPENG) based on soft biocompatible materials (Figure 6j–l). Cardanol oil (CA), as a plasticizer, can form a dense fiber network during the electrospinning process of PVDF, improving the dielectric constant and flexibility of PVDF and endowing the device with ultra-sensitive electromechanical conversion ability [94].

Surface modification technology plays an important role in improving device output. By changing the degree of interfacial friction and dielectric properties of thin films through physical or chemical methods, the device output can be further improved.

## 4. Integrated Strategy in Hybrid Energy Harvester

The integration strategy of hybrid energy harvesting systems is crucial. Through reasonable integration, energy harvesting systems can have the characteristics of efficient harvesting and a wide application range in different environments.

### 4.1. Multi-Source Operation Mode under Complex Conditions

#### 4.1.1. Alternating Operation Strategy

**Figure 7:**
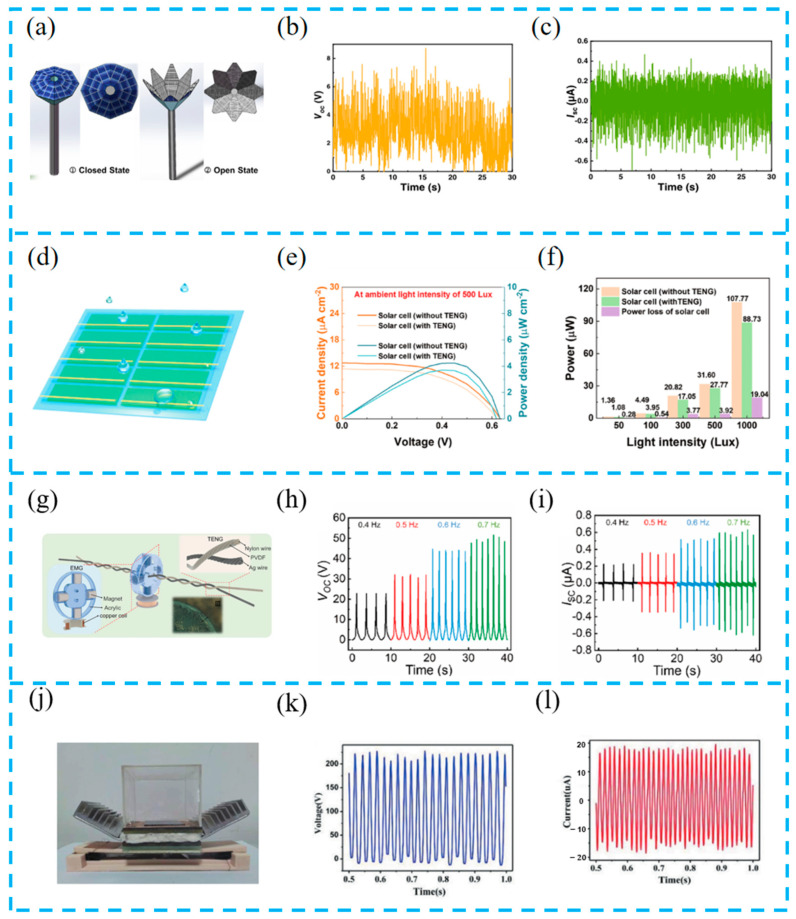
(**a**) Schematic diagram of the smart solar panel umbrella system at closed state under sunny mode and open state under rainy mode; practical V_oc_ (**b**) and I_sc_ (**c**) outputs of the IDE-TENG using a shower connected with a household faucet for simulation of the rain scenario; reprinted from [95] with permission from Elsevier. (**d**) Schematic diagram of the R-TENG array; (**e**) current density (J–V) and power density (P–V) curves of the commercial solar cell unit with/without the R-TENG at ambient light intensity of 500 Lux, which is approximate to the outdoor light intensity in overcast and rainy weather; (**f**) power of the solar cell unit with/without R-TENG and the power loss of the solar cell unit under the influence of R-TENG at different ambient light intensities; reprinted from [96] with permission from Wiley Online Library. (**g**) Structural design and detailed components of the proposed whirligig-HNG; electrical output performance of the TENG at different frequencies of pulling force, which include V_OC_ (**h**), I_SC_ (**i**); reprinted from [97] with permission from Springer. (**j**) The structure of the hybrid generator; (**k**) output voltage and (**l**) current of the TENG part; reprinted from [98] with permission from Elsevier.

The hybrid energy harvesting system, due to its characteristic of collecting multiple energy sources, can operate alternately to compensate for the limitations of single energy harvesting, thereby increasing the harvesting efficiency of the system. For example, Liu et al. used polysilicon solar cells and an interdigitated electrode structure triboelectric nanogenerator (IDE-TENG) to form an umbrella-shaped energy harvesting system (Figure 7a–c). The automatic opening and closing function of the intelligent solar panel system is achieved using microcontrollers and raindrop sensors. The system can work alternately with weather changes, maintaining a high power conversion efficiency of 19.61% of the solar cell, serving as an uninterrupted self power supply system in any weather, effectively saving the use of fossil fuels [95].

Ye et al. designed an efficient R-TENG array (Figure 7d–f). The TENG works effectively on rainy days and the solar panel continues to work on sunny days, forming a complementary solution that achieves energy harvesting and sustainable energy supply throughout the day. In addition, a self-powered wireless light intensity monitoring system was demonstrated for real-time and all-day weather monitoring, and the alternating operation strategy of this system provides a reference for sustainable collection systems [96].

Due to the applicability of EMGs and TENGs in high-frequency and low-frequency applications, Dan et al. adopted a segmented alternating working strategy to expand the energy harvesting range of hybrid devices (Figure 7g–i). The rotating structure in the device can convert low-frequency linear displacement into extremely high-frequency rotational motion. At low frequencies, frictional energy is obtained through the supercoiling/uncoiling process of the device on the pull rope. This clever design validates the possibility of alternating TENG and EMG operation [97].

Liu et al. integrated a TEG and TENG using a converter (Figure 7j–l). This device can independently or simultaneously obtain solar and wind energy from the environment and use them as energy for sensors and wireless transmission devices in the system. Compared with individual TEG and TENG units, the charging voltage of this device increased by 93.3% and 28.9%, respectively [98].

#### 4.1.2. Simultaneous Operation Strategy

**Figure 8:**
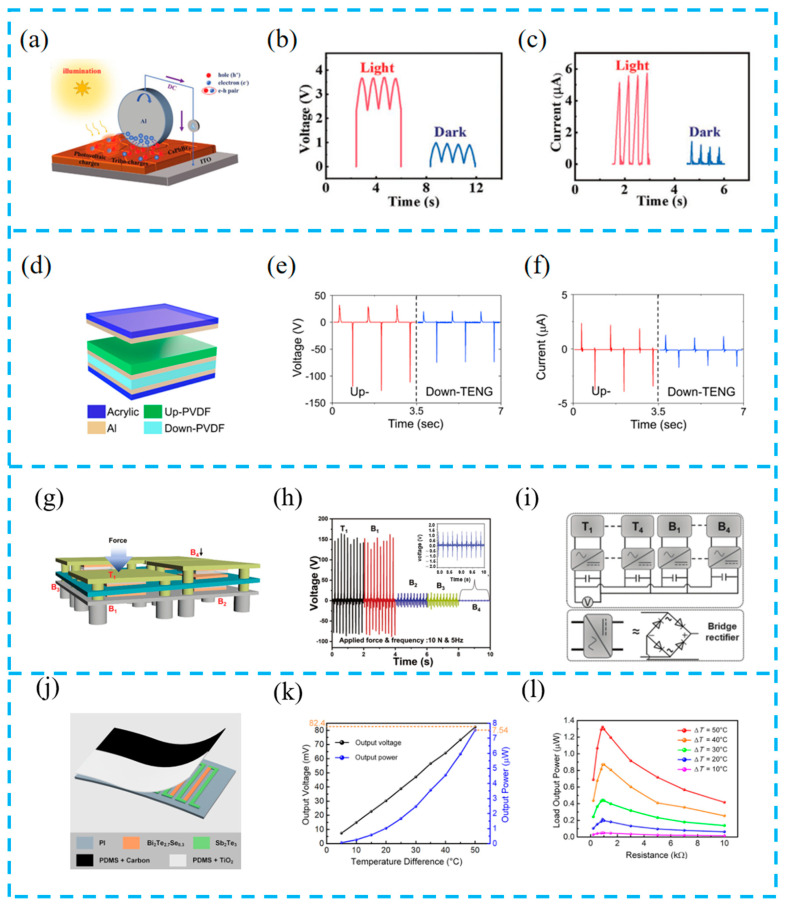
(**a**) Schematic diagram of the Al/CsPbBr_3_ system under sunlight; (**b**) V_OC_ and (**c**) I_SC_ output of the TENG based on a dynamic Al/CsPbBr_3_ Schottky junction under AM 1.5 G sunlight or in a dark environment at a speed of 0.7 ms^−1^; reprinted from [99] with permission from Wiley Online Library. (**d**) A schematic diagram; open-circuit voltage (**e**) and short-circuit current (**f**) of PVDF-based TENG; reprinted from [100] with permission from Elsevier. (**g**) Schematic diagram of the MCHCFS illustrating applied force distribution across different HNGs; (**h**) output voltage from the HNGs within the MCHCFS when a force is applied only on top T1 HNG; (**i**) schematic diagram of the electrical circuit implemented in MCHCFS; reprinted from [101] with permission from Wiley Online Library. (**j**) Film schematic diagram; (**k**) the PTEG thermoelectric open-circuit output voltage and output power increased with the temperature difference and reached 82.4 V and 7.54 μW, respectively, at a temperature difference of 50 °C; (**l**) the dependence of the PTEG thermoelectric output power on various load resistances at different temperature differences indicated that the maximum load output power was 1.32 μW when the temperature difference and load resistance were 50 °C and 0.9 kΩ, respectively; reprinted from [102] with permission from ACS.

The advantage of a hybrid energy harvesting system is that it can adjust the working mode according to needs. The strategy of running the system simultaneously can maximize system output, but it will limit its application scenarios. Yuan et al. prepared a multifunctional DC-TENG that can harvest both mechanical energy and solar energy by constructing a dynamic Al/CsPbBr3 Schottky junction (Figure 8a–c). The device exhibited excellent stability, but its output decreased significantly under dark conditions. This work not only provides a new way to improve working stability through a rolling mode device structure but also implements a strategy for simultaneous device operation [99].

The simultaneous operation strategy adopts a multi-part simultaneous triggering mode, and each part can not only be assembled horizontally but also designed vertically. Lee et al. vertically stacked TENG components on PENG components to prepare a mixed foot mechanical energy harvesting device (Figure 8d–f). When a person is walking normally, both components operate simultaneously, generating enough energy to run the LED. In addition, the device can serve as a self-powered device used to monitor the pressure distribution on the feet. This work provides a method for achieving a safe self-power supply from human health detection systems [100].

Paranjape et al. made a multi-stage continuously connected hybrid nanogenerator (HNG) using composite films (Figure 8g–i) and optimized the electrical output performance of the HNG. Then, it is combined with the floor system (MCHCFS). Due to the vertical multi-layer structure of the device, the friction and piezoelectric parts operate simultaneously to collect mechanical energy during human movement. The collected energy can be used for sustainable road lighting to save huge electricity waste [101].

Wen et al. proposed a flexible hybrid photothermal generator (PTEG) (Figure 8j–l). The generator consists of a TEG and a photothermal conversion layer and is based on a single working mechanism to simultaneously obtain thermal and radiation energy. Through system testing, experiments have demonstrated its significant mechanical reliability and output stability, indicating the feasibility and potential of the developed PTEG as a reliable power source [102].

### 4.2. Multi-Component Coordination Work Strategy

#### 4.2.1. External Circuit Assistance

**Figure 9:**
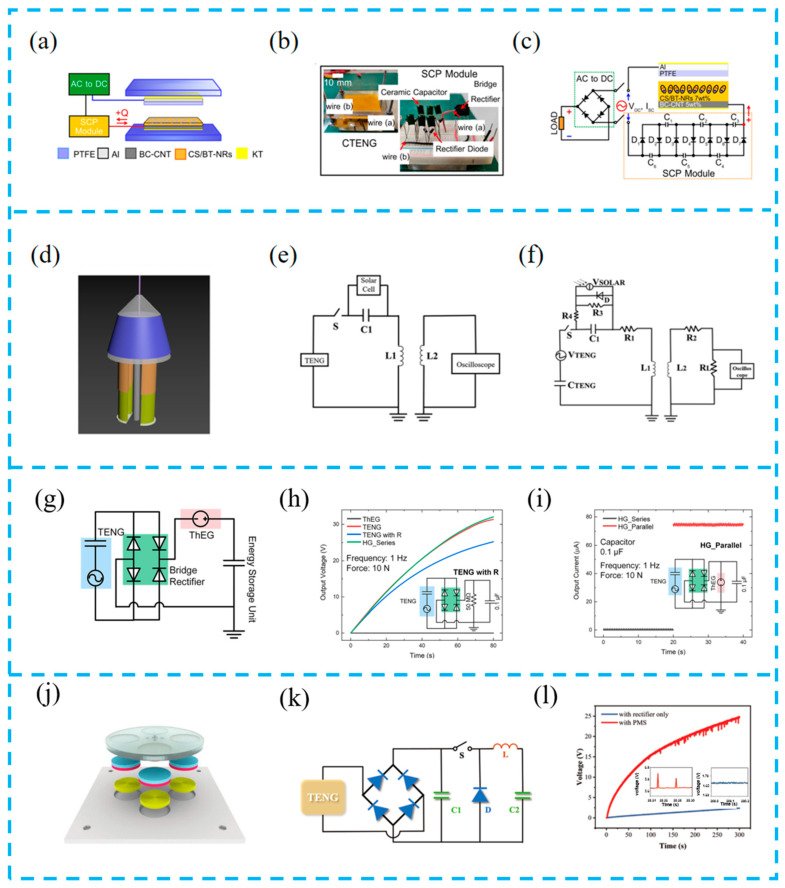
(**a**) Schematic diagram of the internally charged CTENG excitation; (**b**) real CTENG device and electronic components; (**c**) circuit diagram of the internally charged excitation system; reprinted from [86] with permission from Elsevier. (**d**) Structure design; (**e**) schematic diagram of the self-driving wireless transmission system and (**f**) the equivalent circuit of the system; reprinted from [103] with permission from Royal Society of Chemistry. (**g**) Circuit diagrams for charging capacitor; (**h**) capacitor charging curves comparing the output voltage by changing the input conditions of only ThEG, only TENG, TENG with resistor, and hybrid device with series connection; (**i**) output current from the capacitor using hybrid devices with series connection and parallel connection; reprinted from [104] with permission from Wiley Online Library. (**j**) Schematic diagram of the EMG; (**k**) the circuit diagram of the power management system (PMS) for TENG; (**l**) charging curve with a single rectifier and PMS to charge 4700 µF capacitor; reprinted from [105] with permission from Wiley Online Library.

The overall output of the energy harvesting system not only relies on the output performance of hybrid devices but also requires external circuit assistance. Since the output form of a TENG is AC, a rectification circuit is required to convert it into DC to supply power for electronic components. Moreover, with the help of an external circuit, the charging speed can be accelerated and the charge density of the TENG can be improved. For example, the hybrid nanogenerator prepared by Pongampai et al. is combined with the designed self-charge pump (SCP) module to improve the charge density of the friction layer (Figure 9a–c). The SCP module consists of multiple diodes and capacitors connected in parallel. During each contact separation process, the friction layer reversely increases the charge greater than the output of the TENG through the SCP, leading to the internal increase in the ultra-high charge and rapid excitation of the TENG to fulfill system requirements. The power output of the integrated SCP module has increased by more than four times compared to the original chitosan TENGs [86].

The external circuit design generates DC pulses that are conducive to energy transmission. Shi et al. set up specialized mechanical switches to connect the TENG with photovoltaic cells in series (Figure 9d–f). By generating DC pulses through LC oscillation and coupling the receiving coil, the system has a stable wireless transmission capability [103].

By changing connections and adding external components to optimize the charging speed, Kim et al. developed an energy collection system based on a TENG and TEG (Figure 9g–i) and used cyclic voltammetry and discharge time to verify the electrochemical characteristics of the supercapacitor during constant current discharge. The collected energy was then stored in the supercapacitor-driven photosensitive system to provide optical warning signals to animals [104].

Wang et al. developed a high-performance ocean energy harvesting device to ensure the continuous operation of wireless sensor nodes. A power management system (PMS) was designed to improve the charging efficiency of the TENG (Figure 9j–l). The PMS system is composed of a rectifier, capacitor, and other devices. AC is converted into DC, and then the PMS will increase the energy stored in C2 after the recirculation switch S. Experiments have shown that adding the PMS increases the system output by more than 10-fold. A reliable power management strategy can improve system performance and reduce power consumption [105].

#### 4.2.2. Intelligent System Components

**Figure 10:**
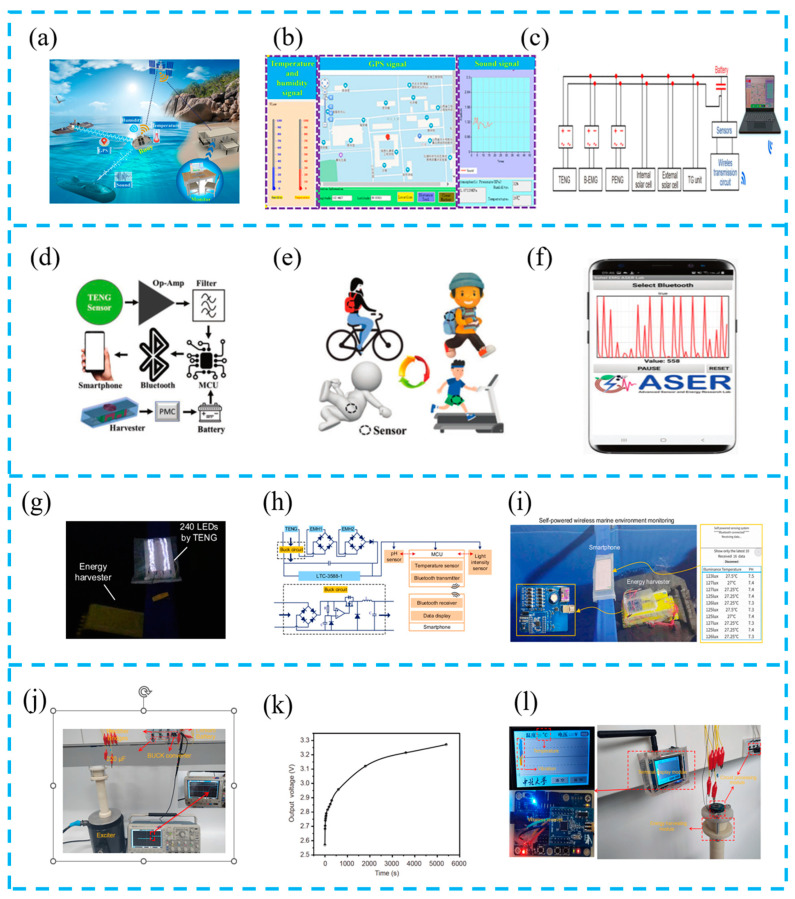
(**a**) Working sketch of the hybrid nanogenerator as a self-powered ocean environment monitoring tool; (**b**) temperature signal, humidity signal, GPS signal, and sound signal tracking using a computer LabVIEW (https://www.21ic.com/ni/labview.html); (**c**) circuit diagram of the self-powered wireless monitoring system; reprinted from [106] with permission from Science press. (**d**) Architecture of a self-powered human motion monitoring system for IoT applications; (**e**) conceptual illustration for monitoring various human motions; (**f**) photograph for demonstrating self-powered wireless human motion; reprinted from [107] with permission from Wiley Online Library. (**g**) Photograph of 240 LEDs lighted directly by the TENG in the MIWEH; (**h**) schematic circuit diagram of the self-powered marine environment monitoring system; (**i**) photograph of self-powered illuminance/temperature/pH value of marine environment monitoring and wireless signal transmission system; reprinted from [108] with permission from Elsevier. (**j**) Charging of a Li-ion battery of 30 mA h; (**k**) charging characteristic curve of the hybrid generator during the charging of a 30 mA h Li-ion battery; (**l**) photograph of the self-powered temperature and vibration wireless monitoring system; reprinted from [109] with permission from Science Press.

Hybrid energy harvesting systems are widely combined with various sensors to form intelligent systems due to their efficient energy harvesting and sensing performance. Feng et al. integrated a hybrid energy harvesting device, power management circuit, sensor, microcontroller, and wireless communication module to create an intelligent ocean buoy (Figure 10a–c). Under the continuous stimulation of waves, this system can send temperature and humidity signals, GPS signals, and sound signals to the receiving end. Based on these signals, outdoor marine environment simulation experiments were conducted, which will provide important assistance in achieving intelligent unmanned monitoring of the ocean in the future [106].

By customizing and developing smartphone applications, better control will be achieved based on hybrid energy harvesting systems. Rana et al. developed an energy harvesting device driven by biomechanics as a continuous power source (Figure 10d–f). Based on this device, an intelligent self-powered wireless human motion monitoring system has been established, consisting of MCU, Bluetooth module, and smartphone. The Bluetooth module wirelessly transmits sensor data to smartphones and customizes smartphone applications with signals. This system can recognize movements such as standing, walking, and running, achieving a fully self-powered wireless human motion monitoring system [107].

In addition, in order to increase the energy conversion efficiency of TENGs, Zhao et al. reported a self-powered wireless marine environment monitoring system (Figure 10g–i), designed a buck circuit, and adopted a power management module to further stabilize electrical energy and reduce energy consumption. In addition, the TENG integrates with various sensors, Bluetooth modules, and microcontrollers, and displays real-time information such as temperature, lighting, pH value, etc., on smartphones. This work demonstrates the possibility of combining intelligent system energy collection with self-powered Internet of Things (IoT) [108].

Xue et al. developed a self-powered wireless temperature and vibration monitoring system based on LTC3331 as the core of an energy harvesting controller (Figure 10j–l). The system consists of an energy harvesting module, a circuit processing module, and a terminal display module. If an abnormality occurs in the monitored object, the system is activated and automatically establishes a connection with the terminal display module through the wireless transmission module to read the current temperature and acceleration signals. At the same time, temperature data are displayed in digital form on the terminal, and vibration data are displayed in waveform form on the terminal. This work is expected to achieve the wireless monitoring of mechanical equipment without an external power supply [109].

## 5. Application of Multifunctional Sensor Based on HEHTNGs

HEHTNGs are widely used as multifunctional sensors due to their multi-energy harvesting characteristics, good adaptability to different environments, and efficient energy conversion efficiency.

### 5.1. Environmental Energy Harvesting

#### 5.1.1. HEHTNGs for Wind Energy Harvesting

**Figure 11:**
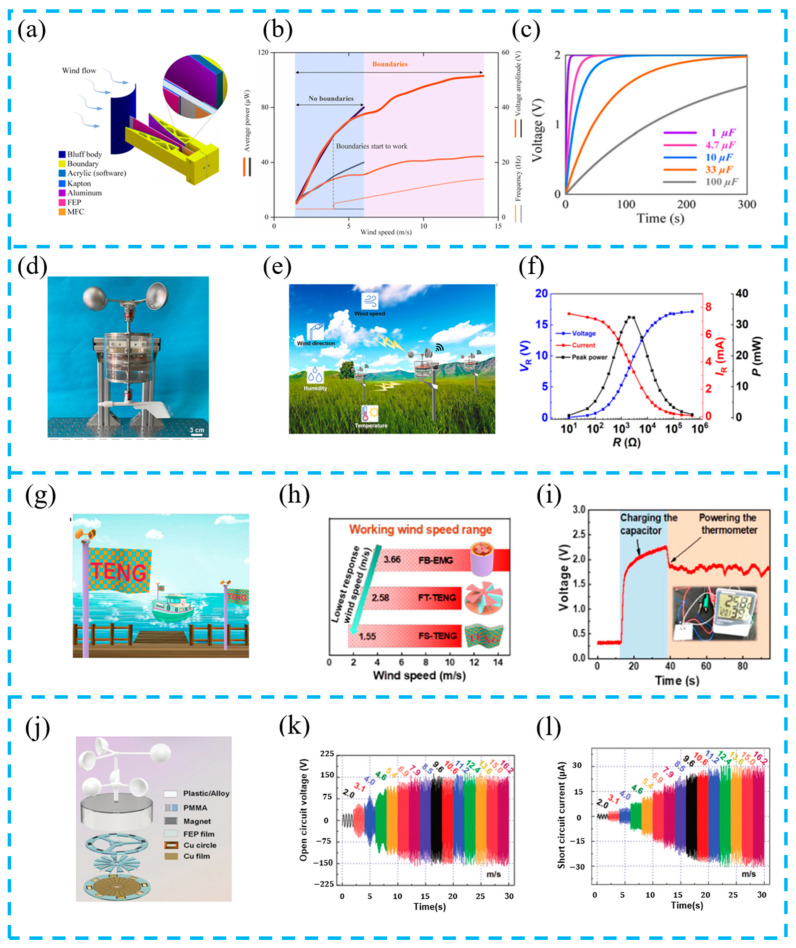
(**a**) Design of a synergetic hybrid piezoelectric–triboelectric wind energy harvester (SHPTWEH); (**b**) comparison of piezoelectric wind energy harvesters with and without boundaries; (**c**) the capacitor charging characteristics of the SHPTWEH with a 14 m/s wind; reprinted from [110] with permission from Elsevier. (**d**) Photograph of ES-ETHG device; (**e**) conceptual diagram of SDWS; (**f**) voltage, current, and the peak power density of EMG change with different load resistance (300 rpm); reprinted from [111] with permission from ACS. (**g**) Application scenario of the FTEHG on a ship and in a port; (**h**) working range of wind speed of the three generators, which indicates that the FS-TENG has the minimum response wind speed; (**i**) demonstration of FTEHG for powering electronic devices; reprinted from [112] with permission from ACS. (**j**) Expanded structural schematic of TEHG. Electrical measurements of the TEHG; (**k**) open-circuit voltage V_oc_; (**l**) short-circuit current I_sc_; reprinted from [113] with permission from Wiley Online Library.

In wind power generation, traditional turbine design and installation have drawbacks such as a high cost and complex equipment, which are not suitable for large-scale applications. However, TENGs can overcome these drawbacks. Many researchers shifted their research direction to TENG-based energy harvesting systems. Wang et al. designed a hybrid nanogenerator (Figure 11a–c). Because of the reasonable design of the system structure, the TENG has a low equivalent stiffness and works effectively at low wind speeds. At high wind speed, it limits the maximum deformation of the beam to avoid damage to the equipment and increases the vibration frequency and conversion efficiency. Experiments have shown that this design improves the conversion efficiency of wind energy to electricity, expands the working range of wind speed, and can be applied to small-scale wind power generation systems in the future [110].

In another work, wind energy harvesting devices are combined with other sensors to form an intelligent agriculture self-powered distributed meteorological sensing system. Zhang et al. proposed this system (Figure 11d–f), which can power sensors and the entire Internet of Things system, as well as remotely collect multiple physical quantities in the environment. The application of this system has increased the development of intelligent agriculture [111].

EMGs and TENGs have good harvesting efficiency in high and low frequencies, respectively. Ye et al. reported a triboelectric–electromagnetic hybrid nanogenerator (FTEHG) (Figure 11g–i). The system has a wide operating range of wind speed (1.55 ms^−1^ to 15 ms^−1^) and can effectively collect various levels of wind energy through an optimized structural design. Experiments have shown that the FTEHG has proven to be a wind speed sensor that can successfully charge a 47 μF capacitor to 1.5 V in 4 s, and is thus a system with great potential for wind energy harvesting and sensing [112].

Hybrid wind energy harvesting devices of EMGs and TENGs are being developed. Yong et al. designed a dual-rotor triboelectric–electromagnetic hybrid nanogenerator (TEHG) through the synergy between TENGs and EMGs (Figure 11j–l), resulting in efficient energy harvesting (41.05 Wm^−3^) at wind speeds of 2–16 ms^−1^. In addition, a dual-channel power management topology (DcPMT) has been established to control the outputs of two modules in the TEHG. This system has the advantages of broadband and efficient wind energy harvesting and can provide 3.3 V voltage to power electronic products, playing an important role in improving the environmental adaptability of the Internet of Things [113]. A summary and comparison of the recently developed hybrid devices are listed in Table 1.

#### 5.1.2. HEHTNGs for Raindrop Energy Harvesting

**Figure 12:**
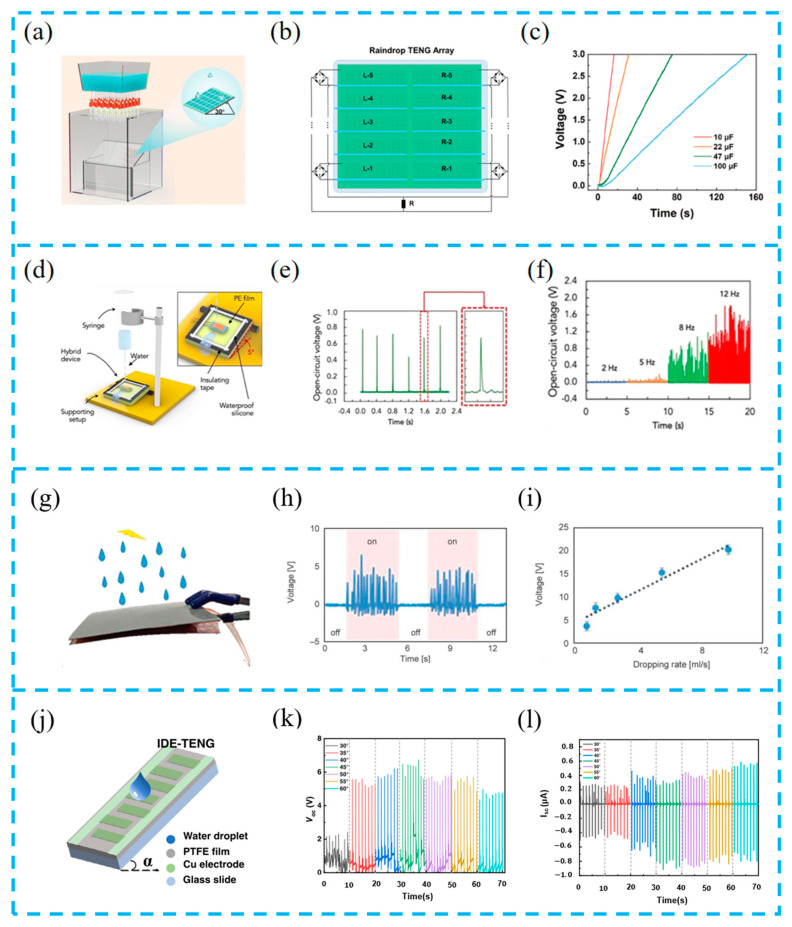
(**a**) Schematic illustration of the arrayed device to simulate actual rainfall; (**b**) arrangement diagram of the common electrode R-TENG array; (**c**) charging voltage on different capacitors charged by the R-TENG array; reprinted from [96] with permission from Wiley Online Library. (**d**) Three-dimensional representation of the custom-made setup for simulating rainfalls; (**e**) the open-circuit voltage of the HNG under impact of single 100 µL water droplets; (**f**) the open-circuit voltage of the HNG under the impact of single raindrops at different frequencies in the range of 2–12 Hz; reprinted from [114] with permission from Elsevier. (**g**) Image of the PTHG fixed to the holder and driven by simulated water dropping; (**h**) the corresponding voltage output of the simulated water dropping in the on and off state; (**i**) the output voltage of PTHG linearly increases from 3.8 ± 0.43 to 20.1 ± 2.1 V with the input of simulated water rate dropping from 1 to 10 mL/s; reprinted from [115] with permission from Express Polymer Letters. (**j**) Device structure diagram; the output voltage (**k**) and current output (**l**) are at different tilt angles; reprinted from [95] with permission from Elsevier.

Many regions have rainy conditions, and the harvesting of raindrop energy is easily overlooked. However, with the development of TENGs, raindrop energy can compensate for SCs’ shortcomings on rainy days, forming a complementary solution. Ye et al. prepared a hybrid nanogenerator array (Figure 12a–c). By systematically optimizing the structure, size, and array distribution density, the overlapping effect generated by multiple raindrops is avoided, thus reducing the probability of the electric signal cancellation of adjacent raindrops, device complexity, and manufacturing cost. In addition, natural rainfall processes were simulated, and highly transparent TENG array surfaces were developed. Finally, a self-powered wireless environmental monitoring system was established for real-time and all-day weather monitoring [96].

The main research on raindrop energy harvesting focuses on two types of energy: raindrop impact energy and electrostatic transfer generated by solid–liquid contact. Mariello et al. designed a multifunctional, flexible, and conformal hybrid nanogenerator (HNG) to collect energy from different water transfer sources (i.e., impact/breakwater, raindrops, buoyancy waves) (Figure 12d–f), greatly increasing the stability of energy harvesting under continuous impacts, especially under strong pulse and raindrop impacts. They also proposed two customized devices to capture wave energy and monitor human motion, demonstrating their versatility as sensors [114].

Chen et al. prepared a fully encapsulated piezoelectric–triboelectric hybrid nanogenerator (PTHG) (Figure 12g–i). On the one hand, the piezoelectric layer used electrospun PVDF, and, on the other hand, the triboelectric layer surface used triangular nano textures to increase the triboelectric performance. By simulating the rain process, it was verified that as the water droplet rate increases, the output voltage value increases proportionally. When the rain rate is 10 mL/s, the maximum output voltage can reach ~20 V, and the area power density can reach ~0.981 mW/m^2^ [115].

Inspired by solid–liquid contact, Liu et al. designed an interdigital electrode structure to integrate solar panels with a TENG (IDE-TENG) (Figure 12j–l). They studied the effects of the friction layer, inclination angle, and various liquid types on system output, and achieved an automatic opening and closing function and high power conversion efficiency of intelligent energy harvesting systems. This system helps to efficiently harvest and utilize clean energy, saving fossil energy consumption [95]. A summary and comparison of the recently developed hybrid devices are listed in Table 2.

#### 5.1.3. HEHTNGs for Sound Energy Harvesting

**Figure 13:**
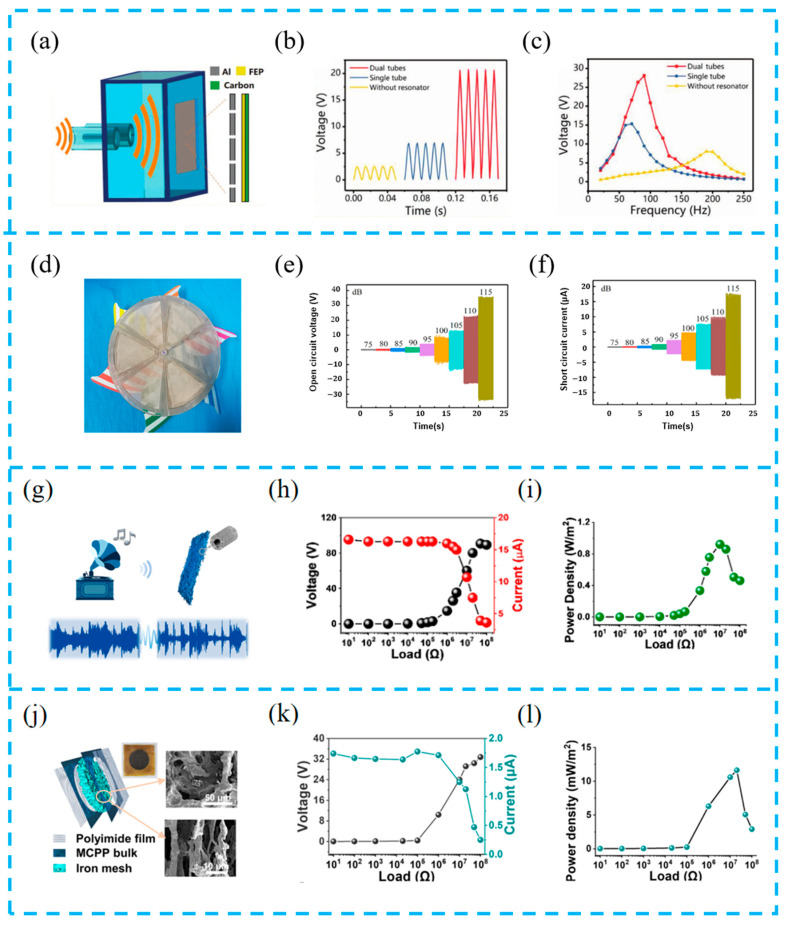
(**a**) Structure scheme of the HR-TENG; (**b**) the open-circuit voltage of the HR-TENGs under the same acoustic wave condition; (**c**) the open-circuit voltage of the HR-TENGs at different acoustic frequencies; reprinted from [116] with permission from Wiley Online Library. (**d**) The front optical images of the hybridized TENGs; (**e**) V_oc_ and (**f**) I_sc_ of the S-TENG with different sound pressures; reprinted from [117] with permission from Elsevier. (**g**) Schematic diagram of the realization from sound to electricity with the fabricated PHVAH; (**h**) output voltages and currents of PHVAH with different external loading resistance; (**i**) areal power densities of PHVAH with different external loading resistances; reprinted from [118] with permission from ACS. (**j**) Structure diagram of the MCPP ANG and SEM images of the aerogel bulk; (**k**) output voltage and current of the MCPP ANG with different external loads; (**l**) output power density of the MCPP ANG with different external loads; reprinted from [119] with permission from Elsevier.

Sound energy can widely exist in the surrounding environment but, due to the low power density of sound waves and the lack of effective sound harvesting technology, it is not systematically researched and developed like other energy sources. The working principle of converting sound energy into electrical energy is the triboelectric effect and electrostatic induction effect of acoustic vibration, so sound energy harvesting devices based on TENGs have been widely studied. Zhao et al. prepared a novel triboelectric nanogenerator (HR-TENG) based on a dual-tube Helmholtz resonator (Figure 13a–c). The device consists of a Helmholtz resonant cavity, a metal film with uniformly distributed acoustic holes, and a dielectric soft film with single-sided printed electrode ink, coupling the sound wave propagation mechanism to improve the device performance output and broaden the frequency [116].

In addition, some researchers have efficiently utilized wind and sound energy. Wang et al. have implemented a hybrid triboelectric nanogenerator (TENG) inspired by windmills to simultaneously capture wind and sound energy (Figure 13d–f). One of the conductive fabric electrodes is shared by the wind-driven TENG (W-TENG) and the sound-driven S-TENG. The unique structural design enables the W-TENG to enhance the electrical output of the S-TENG, which is analyzed and verified through the fast Fourier transform (FFT) of electrical signals [117].

In order to improve the efficiency of harvesting sound energy, a porous three-dimensional structure is designed to increase the triboelectric effect. Yu et al. used nanoporous PVDF hollow fibers and PDMS valves to simulate the tympanic membrane (PHVAH) (Figure 13g–i). The porous structure increases porosity, and the effective combination of piezoelectric and triboelectric material not only recognizes the audio signal but also converts sound into electrical energy, making this hybrid device a promising candidate for acoustoelectric conversion [118].

On the basis of previous studies, Yu et al. designed a unique beam-like structure from a structural design perspective, promoting the diffraction and scattering of sound waves inside the hole wall, enhancing its vibration and friction (Figure 13j–l). In addition, by eliminating the need for contact separation structures or external resonant cavities, this design greatly simplifies the structure and contributes to the flexibility and durability of the device [119]. A summary and comparison of the recently developed hybrid devices are listed in Table 3.

#### 5.1.4. HEHTNGs for Ocean Energy Harvesting

**Figure 14:**
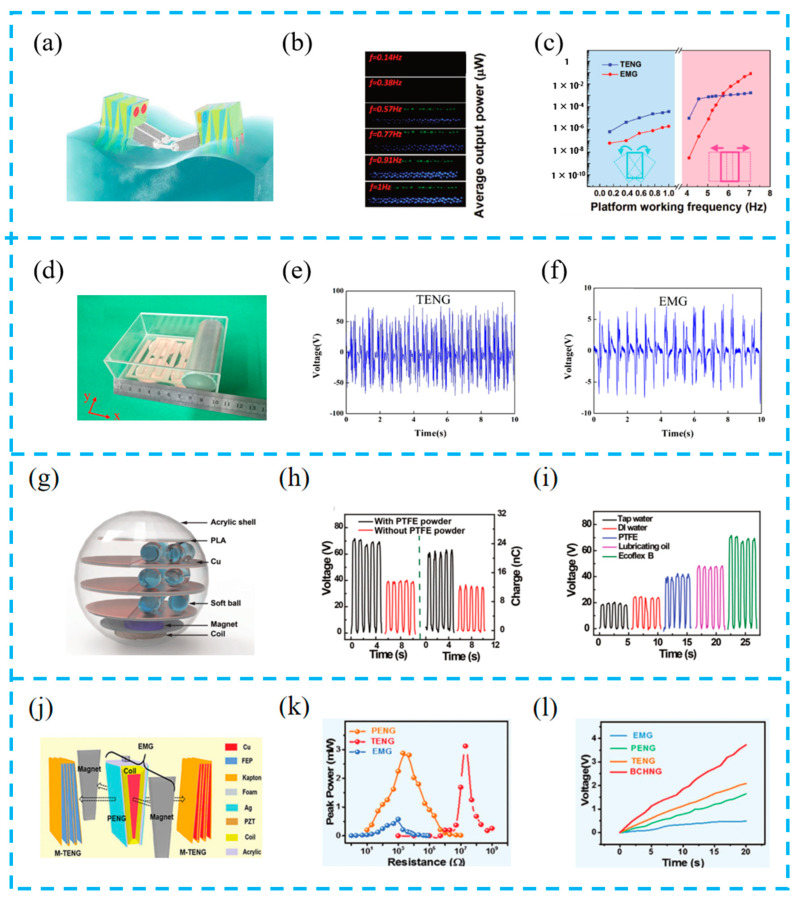
(**a**) Structural design of ocean energy hybrid nanogenerator; (**b**) photographs of LED arrays supplied by TENG and EMG under shaking mode; (**c**) the average output powers for both TENG and EMG under varied platform working frequencies with LED loads; reprinted from [120] with permission from Wiley Online Library. (**d**) Picture of the triboelectric–electromagnetic hybrid nanogenerator. The electrical output performance of TENG and EMG; (**e**) the open-circuit voltage of TENG; (**f**) the open-circuit voltage of EMG; reprinted from [121] with permission from Elsevier. (**g**) Schematic illustration of the concept design for TEHG; (**h**) open-circuit voltage and transferred charge of the SB-TENG using two softballs with and without PTFE powder; (**i**) variation in the induced voltage with the types of the filled liquid; reprinted from [122] with permission from Wiley Online Library. (**j**) Structural components for BCHNG with three kinds of generators; (**k**) the corresponding peak-power–resistance profiles of the BCHNG module with three kinds of generators; (**l**) the charge curve of three kinds of generators and BCHNG modules; reprinted from [123] with permission from Wiley Online Library.

Ocean energy is one of the most abundant sources of energy, and the development of ocean energy is challenging due to the corrosion of electronic devices caused by seawater and the susceptibility to adverse weather conditions on the sea surface. Wang et al. made a hybrid nanogenerator system with an internal topology (Figure 14a–c). Using an independent contact mode, the etched polytetrafluoroethylene film and Cu electrode are designed into a cubic structure, with the TENG and EMG forming mutually compensated outputs. On different platforms, the effects of oscillation frequency, amplitude, and dielectric materials on the output performance of the hybrid system are studied, and the optimal operating frequency range of the system is proved. This work provides an effective method for a hybrid nanogenerator as an energy harvesting device in a wide frequency range [120].

Currently, the research direction of TENG-based ocean energy harvesting mainly includes system design, structural design, and external incentives. Hao et al. designed a box-type energy harvesting device (Figure 14d–f). The contact and separation between the PMMA cylinder wrapped in aluminum film and the Ag electrode wrapped in silicon film result in frictional electrification. The EMG part is a magnetic ball rolling on four copper coils to generate electricity. The experimental results show that the instantaneous maximum output power of the TENG and EMG at a load of 100 MΩ and 1 kΩ is 0.08 mW and 14.9 mW, respectively. In addition, the hybrid nanogenerator can light 60 LED lights at the same time [121].

Encapsulated devices have better corrosion resistance. Pang et al. used silicon shells to prepare soft spherical devices (TEHG) (Figure 14g–i) and studied key parameters for energy collection performance by changing the type of filling liquid, thickness, number of layers, and soft spheres of the silicon shell. This new design and method based on TEHG components for the long-term monitoring of water temperature systems provides a good prospect for constructing a self-powered water sensing system driven by low-frequency water waves [122].

However, inspired by the pendulum, Zhang et al. coupled the dual-pendulum hybrid nanogenerator (BCHNG) module (Figure 14j–l), including a TENG, PENG, and EMG. Not only can these three combinations increase the ability to capture water wave energy, but also the dual-pendulum cone composed of EMG and PENG coils can significantly improve the spatial efficiency of water. It is important to have a precise geometric design and reasonable space utilization, which enable the BCHNG module to achieve a peak power density of 358.5 Wm^−3^. This work provides conditions for the application of high-power self-powered systems [123]. A summary and comparison of the recently developed hybrid devices are listed in Table 4.

### 5.2. Self-Powered Sensors for the Human Body

#### 5.2.1. Wearable Sensors

**Figure 15:**
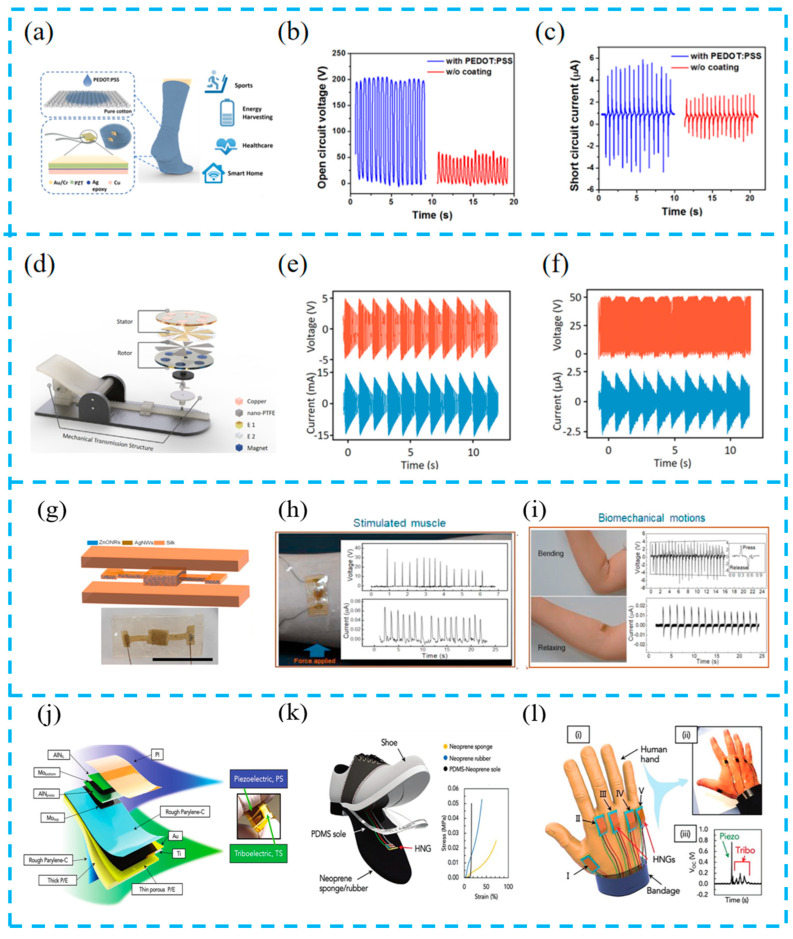
(**a**) Schematic of PEDOT:PSS coated triboelectric S2-sock integrated with PZT force sensors for diversified applications; comparisons of open-circuit voltage (**b**) and short-circuit current (**c**) between PEDOT:PSS-coated sock and original sock; reprinted from [125] with permission from ACS. (**d**) The schematic diagram of the basic structure of wearable non-contact free-rotating hybrid nanogenerator (WRG); the continuous output voltage/current of EMG (**e**) and TENG (**f**) at a 1 Hz frequency load press; reprinted from [126] with permission from Wiley Online Library. (**g**) Schematic and an optical image of the encapsulated hybrid PZ device; energy generation from the EG-skin attached on (**h**) forearm by tapping the hand to stimulate the muscle; (**i**) elbow by bending and releasing; reprinted from [127] with permission from Elsevier. (**j**) Exploded view of the HS with indication of the stacking sequence of layers and of the piezoelectric and triboelectric components; (**k**) 3D representation of the WHS embedded in the PDMS-Neoprene shoe sole; (**l**) application of the WHS for sensing human hand gestures (i) and photo (ii) of a human hand with five WHS sensors attached at its back. (iii) Voltage signal for the full extension of the index finger; reprinted from [128] with permission from Wiley Online Library.

With the rapid demand for sports and fitness, wearable sensors have an important role in the process of the real-time monitoring of human movement status in different ways due to their flexibility, high sensitivity in detecting tiny signals, and so on [124]. Zhu et al. prepared flexible hybrid devices (Figure 15a–c), integrating devices and circuit processing modules into socks to achieve energy harvesting and the sensing of various physiological signals. The experiment showed that, under slight jumps, the load resistance was 59.7 MΩ, and an output power of 1.71 mW was collected from the PEDOT: PSS coating. At the same time, it was verified that this system can be used for real walking pattern recognition and motion tracking in smart home applications [125].

In addition, wearable sensors can not only monitor human motion status but also serve as self-powered devices to power other intelligent sensors, achieving an intelligent integration of IoT devices. Jiang et al. reported a wearable non-contact free-rotating hybrid nanogenerator (Figure 15d–f) that converts the discontinuous gravitational potential energy of the human body into the continuous rotational kinetic energy of the rotor. The unique design can achieve continuous output for more than 2 s under the instantaneous excitation of an external force. Integrating a WRG into shoes, the real-time monitoring of human motion status, and continuous power supply for mobile phones and GPS, this system is expected to be widely used in self-powered systems [126].

With the deepening of research on wearable devices, electronic skins that focus on comfort and practicality have become a research focus. Although devices integrated into clothing can detect human motion status, they still have certain errors and discomfort, and electronic skins can solve such problems. Gogurla et al. have prepared a biocompatible electronic skin (Figure 15g–i). This electronic skin can be realistically and seamlessly integrated with biological tissues and placed in the active parts of the human body to monitor the movement status of the human body and thus determine whether it produces diseases or not. Meanwhile, the results of this research can effectively expand the applications of wearable devices in biomedicine and soft robotics [127].

Mariello et al. reported a hybrid nanogenerator with high sensitivity and super flexibility (Figure 15j–l). The equipment shows high sensitivity and good linearity in different pressure ranges. Due to the contact between the ultra-soft patch and the skin, it can detect micro-triboelectric phenomena and can systematically distinguish gestures and monitor human joint movements. The device can stably and repeatedly recognize typical body movement biological signals, and has good applicability for wearable self-powered sensing systems [128].

#### 5.2.2. Implantable Sensors

**Figure 16:**
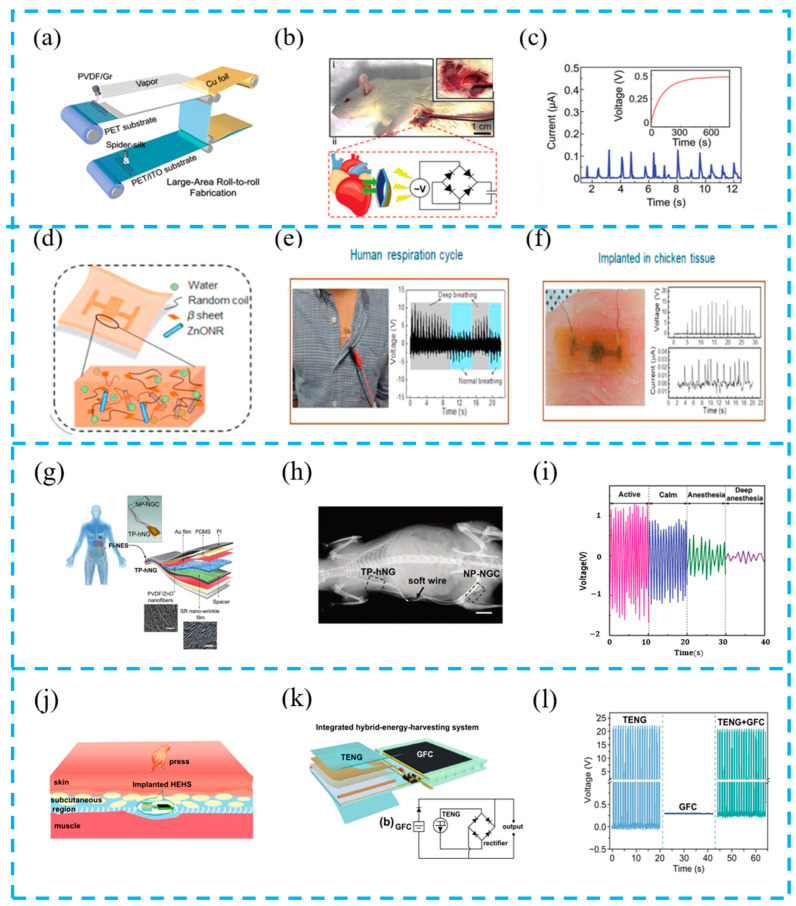
(**a**) Schematic for large-scale and continuous fabrication of TPNG; (**b**) (i) images of a TPNG located in the subdermal chest region at two different scales; (ii) schematic of the energy harvesting process and circuit applied to collect energy from a beating rat heart; (**c**) current signals of implanted TPNG are associated with the beating heart; reprinted from [131] with permission from Wiley Online Library. (**d**) Schematic illustration to show the concept and working principle of artificial EG-skin using silk hydrogel; (**e**) chest for monitoring respiration cycles and (**f**) embedded in chicken breast tissue; reprinted from [127] with permission from Elsevier. (**g**) Structure diagram of FI-NES system; (**h**) small animal X-rays; (**i**) the time curve of the output PSR-ES driven by respiratory motion when SD rats are under different physiological states; reprinted from [132] with permission from Wiley Online Library. (**j**) Conception graph of an implanted HEHS harvesting biomechanical energy and biochemical energy in body; (**k**) structure diagram; (**l**) output voltage of rectified TENG, GFC, and their hybrid device; reprinted from [127] with permission from Springer.

Nowadays, some diseases such as the heart and brain require implantable devices to provide treatment [129]. Implantable devices play an important role in the modern medical field. Because the movement of some organs can be harvested and detected by a nanogenerator as power, the implantable sensing system based on TENGs has been widely studied [130]. Huang et al. prepared a “self-matching” hybrid device based on spider silk protein and PVDF (Figure 16a–c) and designed a “stea-induced phase separation” process to improve the piezoelectric performance. A 1.5 × 1.5 cm sized device was implanted into the heart of mice to monitor and collect the energy of the rat’s heartbeat. After long-term detection, the rats still survived. This flexible and biocompatible sensor can serve as an implanted device for the medical detection and treatment of the human body due to its high energy conversion efficiency [131].

As devices implanted in the human body require better safety, Gogurla et al. used proteins to create an electronic skin (Figure 16d–f). By doping ZnONRs, the piezoelectric properties can be enhanced eight times, and can seamlessly dock with real biological tissues. They were used as artificial tissues for soft robots, not only improving sensor output but also enhancing the safety of the device when used in the human body [127].

The recovery process of nerve injury repair is slow due to its complex and individual differences. Jin et al. developed an implantable system with a physiological adaptation function (Figure 16g–i). The hybrid nanogenerator and nanoporous nerve-guiding catheter are integrated to evaluate the recovery process of long-segment peripheral nerve injury through the level of protein and the shape of the regenerative nerve. For the 15 mm nerve defect, an obvious repair effect is achieved. This work provides an effective method for implanting equipment to treat neurological diseases [132].

Li et al. prepared a TENG-based hybrid energy harvesting system (Figure 16j–l), which is embedded into the body as an implanted device for harvesting bioenergy and biochemical energy. This system allows one unit to be used alone or multiple units to work simultaneously, increasing the flexibility of energy harvesting and improving the output performance, resulting in a higher charging efficiency. Considering the widespread presence of biological and biochemical energy in the human body, the developed system can be used to construct a self-powered health monitoring system in the body [133].

## 6. Conclusions and Perspectives

The TENG-based hybrid energy harvesting system, which couples multiple working mechanisms, can harvest energy in the environment to achieve higher efficiency, multifunctionality and self-sustainability. Combining these characteristics, the integration of TENGs with various energy harvesters in current self-sustaining systems is increasingly attractive, especially in this era of the Internet of Things. It can be seen that HEHTNGs are undergoing fast progress, and current research is mainly focused on the technological improvement process of HEHTNGs from the aspects of device operating principles, performance improvement, and integration concepts. Secondly, HEHTNGs can operate alternately or simultaneously under one or more energy conditions, efficiently collecting energy from different environments, such as wind energy, raindrop energy, ocean energy, and wave energy. And, it can even be used as multifunctional sensors in human health and other environmental stimuli detection while fulfilling the energy needs of electronic devices and self-powered systems. A high power density and sustainability are always the ultimate development trends; in fact, they are also the most active direction of HEHTNGs.

However, there are still some key issues and challenges that need to be addressed, which not only limit the output performance but also limit their potential future applications:

1. Nowadays, most devices are simply stacked and connected, which limits their range of use. Optimized structural designs should be developed to achieve a high power density and sustainability while maintaining device integration and miniaturization to meet various application needs.
2. The durability and stability of devices are easily overlooked, and materials can cause damage in long-term use. Therefore, it is urgent to study materials with self-healing functions.
3. In terms of enhancing performance output, doping and surface modification can increase the output performance of devices, but they can cause material preparation complexity and high costs. Therefore, external excitation methods are sought to promote the generation of functional layer charges to meet the requirements.
4. With the rapid development of the Internet of Things and artificial intelligence, while pursuing a high power density, exploring multifunctionality such as synergistic sensing by integrating devices with each other may achieve novel intelligent features for wearable devices, medical devices, and other environmental monitoring systems.

## Figures and Tables

**Table 1:** Comparison of performance of HEHTNGs in wind energy harvesting.

Hybrid Device Types	Maximum Electrical Outputs		Operating Wind Speed Range	Ref.
PENG + TENG	PENG: ~22.2 V/~103 μW	TENG: ~216 V/~70.3 μW	0–15 m/s	[110]
EMG + TENG	EMG: ~27 V/~11.6 mA/~32.4 mW	TENG: ~13 V/~164 nA	3–15 m/s	[111]
EMG + TENG	EMG: ~4.3 V/~19.5 mA/~4.23 mW	TENG: ~331 V/~18 μA/~2.7 mW	1.55–15 m/s	[112]
EMG + TENG	EMG: ~4.4 V/~8.7 mA/~12 mW	TENG: ~300 V/~30 μA/~5.2 mW	2–16 m/s	[113]

**Table 2:** Comparison of performance of HEHTNGs in raindrop energy harvesting.

Hybrid Device Types	Maximum Electrical Outputs	Inclination Angle	Rain Speed	Ref.
SC + TENG	R-TENG: ~40.80 mW/m^2^	30°	50 mL/min	[96]
PENG + TENG	HNG: ~2.2 V/~9 mW/m^2^	0°	3.3 mL/s	[114]
PENG + TENG	PTNG: ~20 V/~0.981 mW/m^2^	/	10 mL/s	[115]
SC + TENG	IDE-TENG: ~4 V/~8.7 mA/~0.24 mW/m^2^	45°	/	[95]

**Table 3:** Comparison of performance of HEHTNGs in sound energy harvesting.

Hybrid Device Types	Maximum Electrical Outputs	Optimal Frequency	Sound Pressure	Ref.
Helmholtz Resonator +TENG	HR-TENG: ~132 V/~32 µA/~1.82 WPa^−1^m^−2^	70 Hz	88.4 dB	[116]
PENG + TENG	S-TENG: ~80 V/~19 μA/~0.5 mW	180 Hz	115 dB	[117]
PENG + TENG	PHVAH: ~105.5 V/~16.7 μA/~0.92 Wm^−2^	150 Hz	117.6 dB	[118]
PENG + TENG	IDE-TENG: ~34.4 V/~1.74 μA/~11.62 mWm^−2^	150 Hz	115 dB	[119]

**Table 4:** Comparison of performance of HEHTNGs in ocean energy harvesting.

Hybrid Device Types	Maximum Electrical Outputs			Operating Frequency	Ref.
EMG + TENG	EMG: ~1.7 V/~5.4 mA	TENG: ~400 V/~15.3 µA		1 Hz	[120]
EMG + TENG	EMG: ~8.5 V/~0.63 mA	TENG: ~55.6 V/~1 µA		1 Hz	[121]
EMG + TENG	EMG: ~3 V/~15 mA	TENG: ~450 V/~2 µA		2 Hz	[122]
EMG + PENG + TENG	EMG: ~0.65 V/~2.2 mA	PENG: ~11 V/~6 mA	TENG: ~650 V/~150 µA	/	[123]

## Data Availability

Not applicable.
